# Assessing Chronic Pain Among Adults Diagnosed with Diabetes Residing in Rural Appalachia

**DOI:** 10.13023/jah.0603.07

**Published:** 2024-10-01

**Authors:** Brittany L. Smalls, Adebola Adegboyega, Courtney Ortz, Ellen Combs, Tofial Azam, Philip M. Westgate, Nancy Schoenberg

**Affiliations:** University of Kentucky; University of Kentucky; University of Kentucky; University of Kentucky

**Keywords:** Appalachia, chronic condition, cross-sectional, multimorbidity, rural

## Abstract

**Introduction:**

Appalachian populations have some of the highest rates of overdose and comorbidity, all of which are considered risk factors for and contributors to chronic pain.

**Purpose:**

The purpose of this study was to examine the associations of comorbidity, disability (physical limitations), and depression with chronic pain among a community-based sample of Appalachian adults living with diabetes.

**Methods:**

This study used baseline data to conduct a secondary analysis of cross-sectional data (n=356). Data included sociodemographic, disability (physical limitations), chronic pain, and depression measures. These data were collected and analyzed from 2017–2019. Multiple logistic regression was used to investigate the association between comorbidity, disability, depression, and chronic pain.

**Results:**

Participants were predominantly non Hispanic white (98.0%), women (64.6%), and had a mean age of 64.2 years. Comorbidity (*p*=.044), physical limitations (*p*p

**Implications:**

Chronic pain affects physical and psychosocial health among those diagnosed with diabetes who live in rural Appalachian communities. Alleviating chronic pain could have a synergistic benefit to healthy functioning.

## INTRODUCTION

According to the National Institutes of Health, chronic pain is one of the most common complaints across the global healthcare system.^1^ In the United States, chronic pain, described as pain that has persisted for more than three months, affects approximately 100 million adults and is associated with significant emotional distress and/or functional disability, and not attributable to another condition.^2^ Chronic pain constitutes a significant burden within vulnerable, resource-limited communities like Appalachia. Increasingly linked with serious illness, chronic pain intersects with substance use and poses a significant risk factor for Appalachian residents,^3^,^4^ who have twice the national overdose mortality rate.4^5^ Moreover, chronic pain is thought to be more prevalent in the Appalachian Region due to the higher rates of drug use, comorbidities, and lower socioeconomic status.^3^

The escalation of chronic pain among the Appalachian population is often due to occupational exposure and comorbidities. Many of the jobs in rural Appalachia involve potentially unsafe environments and physical danger, including mining, mechanics, and agriculture. All of these jobs use heavy machinery, further increasing the possibility for injuries.^6^ Moreover, some of the comorbidities in the resource-limited Appalachian communities include type 2 diabetes mellitus (T2DM), sleep disturbances, depression and other mental health conditions, physical limitations, and polysubstance use.^7^,^8^ Chronic pain is both a stress state and one of the critical factors that can lead to depression. Their coexistence tends to further aggravate the severity of both disorders.^9^ Epidemiologic studies have shown that the risk of depression for individuals with chronic pain is 2.5 to 4.1 times higher than of those without chronic pain.^10^ Furthermore, depression is one of the primary comorbidities associated with T2DM, where individuals with T2DMs are twice as likely to have coexisting depression.^11^ Depressive symptoms can lead to a diminished ability for self-care, which is associated with a decline in T2DM-related health outcomes.^11^ Interestingly, women, when compared to men, experience greater pain, greater pain-related distress, and show heightened sensitivity to experimentally induced pain.^12^

Various pain syndromes are associated with T2DM and contribute to its morbidity. These pain syndromes affect patients’ quality of life^13^ and lead to increased distress.^14^ For example, diabetic neuropathy is a painful and debilitating disorder that occurs in nearly 20% of T2DM patients.^13^ Symptoms of diabetic neuropathy include burning, electric shocks, stabbing, and pins and needles sensations.^14^ Other types of chronic non-neuropathic pain syndromes, such as frozen shoulder and abdominal pain, are also reported among individuals living with T2DM.^15^

Overall, this description of chronic pain, its association with T2DM, and its multitude of contributing factors show the growing need to better understand how these conditions and factors align. Therefore, the purpose of this study is to evaluate the association between comorbidities, physical limitations, and depression on chronic pain in adults diagnosed with T2DM who live in Appalachia. By understanding this relationship, healthcare professionals and researchers will be able to provide appropriate care and support further research, as it relates to chronic pain, in this population.

## METHODS

### Study Overview

This paper reports secondary analysis of baseline cross–sectional data collected as part of an ongoing study. The baseline data collection included a diverse array of behaviors (e.g., self-care behaviors) and psychosocial factors (e.g., depression) relevant to glycemic control.^16^ Data were collected and analyzed from 2018 to 2019. Study approval was obtained by the Office of Research Integrity at an academic medical center.

### Recruitment

Participants were recruited through community sites within six counties in rural Appalachian Kentucky, including churches, community centers, and senior centers. Community recruitment offers advantages over clinical recruitment, including enrolling hard to reach individuals with impeded access to clinics, avoiding selection bias of healthier participants better able to access clinics, increasing comfort and trust of participants, and ensuring that the facilities are accessible to participants after hours. Participants were eligible to participate if they were 18 years or older, lived in Appalachian Kentucky — determined by Rural-Urban Commuting (RUCA) codes — with no plans to relocate out of the area within the next 18 months, showed a willingness and ability to participate (i.e., no major cognitive impairment), and had a diagnosis of T2DM and/or HbA1c levels ≥ 6.5%. Individuals interested and potentially eligible were asked to complete written consent and undergo point-of-care HbA1c testing to confirm eligibility.

### Measures

#### Demographic variables

The following variables were included as covariates: age, sex, race/ethnicity, marital status, education level, employment status, insurance status (insured v. uninsured), and financial status. The number of chronic conditions was determined using the following question from the Behavioral Risk Factor Surveillance System^17^: “Has a doctor, nurse, or other health professional ever told you that you had any of the following?” The responses included heart attack, coronary heart disease/angina, stroke, kidney disease, high blood pressure, high cholesterol, type 1 diabetes (T1D), and Hepatitis C. Thus, comorbidity was measured by the number of chronic conditions chosen from this list.

#### Physical limitations

Physical limitations were measured with items taken from the SF-36, a 36-item short-form health survey that the participant can rate his/her limitation on a scale of zero to 100, with zero indicating having the most physical limitations.^18^ The questions asked about daily activities (e.g. “Does your health now limit you in these activities? If so, how much?”). Specific activities included vigorous activities (e.g., running), moderate activities (e.g., pushing a vacuum), lifting or carrying groceries, climbing several flights of stairs, bending/kneeling, walking more than a mile, walking several blocks, walking one block, and bathing or dressing themselves.

#### Chronic pain

Chronic pain was measured by using the following question taken from the National Health and Nutrition Examination Survey: “During the past month have you had a problem with pain that lasted more than 24 hours?” If participants responded yes, they were asked, “For how long have you experienced this pain?”^19^ Chronic pain is defined as pain experienced for at least three months^2^; thus, responses were dichotomized into more than three months and less than three months for analysis. The location of pain was assessed using the following question: “Please tell me all areas of your body where you have experienced persistent pain in the past two months.” The options were head, face/mouth, shoulder/arm/hand, neck/back/buttocks, leg/foot, chest/stomach, don’t know, and decline.

#### Depression

We used the Center for Epidemiologic Studies Depression (CES-D) Scale^16^ to measure depressive symptoms. The CES-D scale includes 20 items that assess aspects of depression symptoms such as depressed mood, feelings of guilt and worthlessness, helplessness and hopelessness, and sleep disturbance. Responses use a 4-point Likert scale: 0 (rarely, less than 1 day), 1 (some of the time, 1 to 2 days), 2 (a moderate amount of time, 3 to 4 days), or 3 (most or all of the time, 5 to 7 days). A total depression symptoms cutoff score was utilized such that participants with a CES-D score ≥9 were categorized as depressed and all others were categorized as not depressed.

### Data Analysis

We used frequency distribution and central tendency statistics to summarize participants’ demographics and breakdown of the physical location of where participants reported experiencing pain. To account for the possibility of clustering due to the study design, corresponding *p*-values are obtained from fitting GEE-type marginal logistic regression models. To ensure valid inference, bias-corrected standard errors were utilized.^20^ Models adjust for the demographic variables. To obtain standardized beta coefficients, continuous predictor variables such as age and physical limitation scores are centered and standardized. Model 1 evaluated co-morbidity, physical limitations, and depressive symptoms in three separate adjusted models. Then, in Model 2, a single adjusted model was fit with all three of these potential predictors. Lastly, for Model 3, backward elimination at the 0.05 level was used to assess the most important predictors. All tests were two-sided and statistical significance is defined as *p*<.05. Analyses are conducted in SAS version 9.4.^21^

## RESULTS

Table 1 displays characteristics of the study population. The mean age of participants was 64.2 years (± 10.6). The majority of participants were women (64.6%), married (58.4%), non-Hispanic white (98%), insured (98%), and nonsmokers (89.6%). More than one-third of the population had completed at least a high school education (32.3%) and were retired (42.1%). In addition, most participants reported no depressive symptoms (69.4%) or chronic pain (68.8%). For the nearly one-third who reported chronic pain (n=138), shoulder/arm/hand (45.6%), neck/back/buttocks (73.9%), and leg/foot (65.5%) were the most frequently reported problem locations (Table 2). It should be noted that participants could indicate more than one area where they experienced chronic pain during data collection. As for physical limitations, participants reported an average of two physical activities (± 0.6) they regularly struggled to complete.

Table 3 shows that comorbidity (*p*=0.044), physical limitations (*p*<.001), and depressive symptoms (*p*<.001) are all significantly associated with chronic pain. Regarding co-morbidity, 39.56% (36 of 91) of participants reporting more than two health conditions had chronic pain, relative to 27.82% (69 of 248) of participants reporting two or fewer health conditions, corresponding to a 42% increase in risk of chronic pain. Also, 51.69% (46 of 89) of participants who reported more depressive symptoms had chronic pain, relative to 20.73% (51 of 246) of participants who had fewer depressive symptoms, corresponding to almost a 250% increase in risk. Finally, the mean (SD) physical limitations score was 1.85 (0.50) and 2.23 (0.55) for those with and without chronic pain, respectively, corresponding to an effect size of approximately 0.72, indicating greater limitations in performing daily activities for participants with chronic pain.

Additional factors associated with chronic pain included age (*p*=0.016), employment status (*p*=0.010), and smoking status (*p*=0.005). Participants with chronic pain tended to be younger, having a mean age of 61.74 (SD=9.98) relative to a mean age of 65.16 (SD=10.77) for those without chronic pain. Current smokers were 76% more likely to have chronic pain. Specifically, 50% (18 of 36) of current smokers experience chronic pain, whereas only 28.39% (90 of 317) of non-smokers experience chronic pain. Furthermore, participants who were fully employed were most likely to have chronic pain (34 of 73; 46.58%), whereas those who indicated physical limitations were least likely to have chronic pain (7 of 61; 11.48%).

When adjusting for demographic variables, co-morbidity was no longer a significant predictor of chronic pain (*p*=0.5, Model 2), although physical limitations (*p*<.001) and depressive symptoms (*p*=0.002; **Table 4**) were associated with chronic pain. In summary, a one-unit increase in physical limitations resulted in an adjusted reduction in the estimated odds of chronic pain by 51% (OR=0.5, 95% CI: 0.3, 0.7). Furthermore, greater depressive symptoms increased the adjusted odds of experiencing chronic pain (OR=2.7, 95% CI: 1.5, 4.9).

## DISCUSSION

Approximately one-third of the sample population reported having consistent pain in at least one area of their body for more than three months. Experiencing chronic pain was associated with having comorbidities. For example, having more than two chronic conditions significantly increased the risk of experiencing chronic pain. Notable comorbidities included struggling to complete at least two physical activities and experiencing depressive symptoms, which presented an alarming 250% increased risk of reporting chronic pain. Interestingly, in this sample, chronic pain was primarily reported in three areas of the body — shoulder/arm/hand, neck/back/buttocks, and leg/foot. These findings present insight into how chronic pain affects physical and mental health among those diagnosed with T2DM who live in rural Appalachian communities.

Diabetic neuropathy affects individuals in a variety of ways including loss of sensation, loss of balance, severe pain, and foot ulcers and amputations.^14^ However, more recent literature has indicated that there is a relationship between back and neck pain and those diagnosed with T2DM.^22^ Research shows that intervertebral disk degeneration triggers low back pain and is common in adults with T2DM and unhealthy weight.^23^ When compared to patients without T2DM, those with T2DM had lower improvement in leg pain and lower increases in walking capacity,^24^ but no difference in back pain. In addition, severe pain is independently and inversely associated with being physically active.^25^

Prior research suggests that depressive symptoms and T2DM have a bidirectional relationship that ultimately affects T2DM-related clinical outcomes.^11^ Similarly, chronic pain and depression have been linked.^26^ Hence, it is not surprising that these three conditions show highly significant relationships in this current study. Still, the magnitude of this relationship among this population is remarkable.

Chronic pain itself can be debilitating. This study found that the mean physical limitations score was lower for those with chronic pain as compared to those without, and 11.48% of those who indicated physical limitations were least likely to have chronic pain. Unfortunately, a limitation of this study is that it does not include questions that could determine if the pain experienced by participants was linked specifically to their disability status (physical limitations). Future studies should aim to address this directly, which could lead to a better interpretation of the current studies results. Specifically, questions addressing the relationships between chronic pain, physical limitations, and type of employment would be beneficial. Taken together, we can cautiously infer that chronic pain could be debilitating in T2DM populations. Further research with more precise questioning surrounding this subgroup of participants is needed for additional clarification.

### Limitations

One limitation to the current study is that other comorbid chronic conditions such as cancer, arthritis, and COPD were not included in the study. These conditions contribute to morbidity and mortality. Adding them would enable a more holistic understanding of how comorbidities contribute to chronic pain.

Another limitation of the current study is based on how the question regarding location of pain was asked. The current study is not able to tease out how many participants specifically reported chronic buttock pain. It would be interesting to know how many participants experienced specifically chronic buttock pain, as there is no literature that discusses the relationship or potential causes for chronic buttock pain among those diagnosed with T2DM, including those living in rural Appalachia.

## CONCLUSION

These findings reinforce the extensive body of research that Appalachian residents experience challenges to achieving optimal health and wellbeing. This population is largely geographically isolated with few resources that facilitate self-care activities to reduce their risk of morbidity and mortality. These limitations include but are not limited to walkability/accessible areas to participate in physical activities, access to fresh produce/healthy foods, and affordability of medications, glucometer, and lancets to manage blood sugar, most of which are associated with diabetes complications and pain.

It should also be noted that the mean age of the study sample was 64.2 years (± 10.6). This is of particular importance because public health initiatives are campaigning for older adults to age in place. However, if aging in place results in disability and debilitating chronic pain, older adults may lose their independence. Therefore, it is important that future research considers how to address chronic pain in those with T2DM in rural Appalachia while also accounting for the unique circumstances experienced by older adults who live in those communities.

**SUMMARY BOX**

**What is already known about this topic?**

Appalachian populations have some of the highest rates of overdose and comorbidity, all of which are considered risk factors for and contributors to chronic pain.

**What is added by this report?**

Physical limitations, depression, comorbidity, and demographic variables all have an influence on chronic pain in individuals with T2DM.

**What are the implications for future research?**

It is important that future research considers how to address chronic pain in those suffering with T2DM in rural Appalachia while also accounting for the unique circumstances experienced by older adults who live in those communities.

## Figures and Tables

**Table 1:** Participant characteristics (N=356)

Variable	Mean (± SD) or N (%)
**Age**	64.2 (± 10.6)
**Sex**	
Female	230 (64.6%)
Male	126 (35.4%)
Missing	0 (0%)
**Marital Status**	
Married	208 (58.4%)
Divorced	55 (15.4%)
Never married	21 (5.9%)
Widowed	68 (19.1%)
Missing	4 (1.1%)
**Education**	
HS/GED	115 (32.3%)
Associate’s degree	43 (12.1%)
Bachelor’s degree	24 (6.7%)
Graduate/professional degree	113 (31.7%)
Some college	61 (17.1%)
Missing	0 (0%)
**Employment**	
Full-time	63 (17.7%)
Disability	73 (20.5%)
Homemaker	49 (13.8%)
Part-time	12 (3.4%)
Retired	150 (42.1%)
Unemployed	9 (2.5%)
Missing	0 (0%)
**Race**	
White	349 (98.0%)
Black	7 (2.0%)
Missing	0 (0%)
**Perceived Financial Status**	
Just about enough to get by	154 (43.3%)
More than you need to live well	91 (25.6%)
Sometimes struggle to make ends meet	103 (28.9%)
Missing	8 (2.2%)
**Insurance Status**	
No	7 (2.0%)
Yes	349 (98.0%)
Missing	0 (0%)

**Table 2:** Participants’ self-reported health status (N=356)

Variable	Mean (± SD) or N (%)
**Smoke Status**	
No	319 (89.6%)
Yes	37 (10.4%)
Missing	0 (0%)
**# Of Health Conditions**	
0	17 (4.8%)
1	72 (20.2%)
2	161 (45.2%)
3	58 (16.3%)
4	24 (6.7%)
5	7 (2.0%)
6	2 (0.6%)
Missing	15 (4.2%)
**Depressed (CESD ≥9)**	
No	247 (69.4%)
Yes	90 (25.3%)
Missing 19 (5.3%)	
**Disability (# of Physical Limitations)**	2.12 ± 0.56
**Any Chronic Pain**	
No	245 (68.82%)
Yes	108 (30.34%)
Missing	3 (0.84%)
**Location of Pain**	
Hands	19 (13.77%)
Face/Mouth	7 (5.07%)
Shoulder/Arm/Hand	63 (45.65%%)
Neck/Back/Buttocks	102 (73.91%%)
Leg/Foot	89 (64.49%)
Chest/Stomach	19 (13.77%)
